# Suboptimal outcomes of group III paediatric genitourinary rhabdomyosarcoma-experience from treatment with a multimodal protocol in low- and middle-income setting

**DOI:** 10.3332/ecancer.2025.2049

**Published:** 2025-11-28

**Authors:** Annesha Chakraborti, Badira Cheriyalinkal Parambil, Venkata Rama Mohan Gollamudi, Maya Prasad, Siddhartha Laskar, Nehal Khanna, Jifmi Jose Manjali, Sajid Qureshi, Mukta Ramadwar, Poonam Panjwani, Akshay Baheti, Vasundhara Patil, Sneha Shah, Girish Chinnaswamy

**Affiliations:** 1 Division of Paediatric Oncology, Tata Memorial Hospital, Homi Bhabha National Institute (HBNI), Mumbai 400012, India; 2 Department of Radiation Oncology, Tata Memorial Hospital, Homi Bhabha National Institute (HBNI), Mumbai 400012, India; 3 Department of Paediatric Surgery, Tata Memorial Hospital, Homi Bhabha National Institute (HBNI), Mumbai 400012, India; 4 Department of Pathology, Tata Memorial Hospital, Homi Bhabha National Institute (HBNI), Mumbai 400012, India; 5 Department of Radiodiagnosis, Tata Memorial Hospital, Homi Bhabha National Institute (HBNI), Mumbai 400012, India; 6 Department of Nuclear Medicine, Tata Memorial Hospital, Homi Bhabha National Institute (HBNI), Mumbai 400012, India; 7 Division of Paediatric Oncology, Tata Memorial Hospital, Homi Bhabha National Institute (HBNI), Mumbai 400012, India; a https://orcid.org/0000-0001-6249-9933; b https://orcid.org/0000-0001-6459-2058; c https://orcid.org/0000-0003-0127-7987; d https://orcid.org/0000-0001-8558-4225; e https://orcid.org/0000-0002-3613-687X; f https://orcid.org/0000-0002-6770-5887; g https://orcid.org/0000-0002-4177-2547

**Keywords:** paediatric, genito-urinary, rhabdomyosarcoma, outcomes, prognostic factors, unfavourable site

## Abstract

Genitourinary-Rhabdomyosarcomas (GU-RMS) are challenging to treat due to the probable lifelong sequelae of local therapy. Western-world data show 3-year event-free survival (EFS) and overall survival (OS) of 77% and 86%, respectively, for localised disease, with dismal outcomes for metastatic disease. We studied the clinical profile, outcomes and prognostic factors of GU-RMS treated with a multimodal protocol. Treatment-naïve children ≤ 15years with biopsy-proven GU-RMS treated from January 2013 to June 2022 were retrospectively analysed. Local therapy performed at 10–12 weeks of induction was radiotherapy (RT) and/or surgery. Fifty-two patients with a median tumour size of 5.5 cm (range, 3.4–9.2 cm) were analysed. Four patients (7.8%) had alveolar histology. The bladder was the commonest site of primary (36.5%). Group distribution: I-7 (13.4), II-1 (1.9%), III-35 (67.3%) and IV-9 (17.3%). Local therapy was surgery in 11 (21.5%), RT in 25 (49%) or both in 14 (26.9%) patients. With a median follow-up of 56 months (95% confidence interval (CI): 49.1%–63.1%), 4-year EFS for groups I–IV, were 100%, 50% (95% CI: 41%–59%) and 33.3% (95% CI: 2.6%–64%) (*p* = 0.01), respectively. The corresponding 4-year OS were 100%, 72% (95% CI: 56.4%–87.6%) and 33.3% (95% CI: 2.6%–64%) (*p* = 0.007), respectively. Relapses were locoregional-4 (7.7%), metastatic-5 (9.6%) and combined-4 (7.7%). Tumour size > 6.45 cm significantly affected outcomes in the localised cohort (hazard ratio = 4.1, 95% CI: 1.38–12.1, *p* = 0.01). Outcomes of group III GU-RMS in children treated on a multimodal protocol in our study are suboptimal compared to those from co-operative group trials, probably affected by large tumours at presentation, warranting alternative strategies for optimisation of survival.

## Introduction

Rhabdomyosarcoma (RMS) accounts for 3.5% of cancers in individuals below 14 years of age [1]. Within this subset, Genitourinary-RMS (GU-RMS) constitutes 15%–20% of cases, primarily involving the bladder (B), prostate (P) and paratesticular areas, with occasional occurrences in the vagina, uterus, kidney or ureter [2–4]. Notably, non-bladder, non-prostate and non-kidney primaries demonstrate favourable outcomes [1]. Contemporary treatment protocols for RMS rely on a multimodal approach, which integrates induction (neo-adjuvant) chemotherapy followed by surgery (where feasible) to achieve negative margins while preserving organ function, with or without radiotherapy (RT) [3, 5]. Optimal local control for GU-RMS poses significant challenges due to the anatomical complexity of these regions. Consequently, management often involves lifelong morbidities such as urinary diversions, infertility and erectile dysfunction [3]. Despite these hurdles, high-income countries report 5-year event-free survival (EFS) and overall survival (OS) rates exceeding 76% and 80%, respectively [3]. However, metastatic GU-RMS continues to have a poor prognosis globally, with a 3-year OS of less than 40% [2].

Approaches to local control differ between cooperative groups. The International Society of Paediatric Oncology (SIOP) advocates selective omission of local therapy in specific scenarios, such as vaginal or uterine RMS achieving complete remission with chemotherapy, based on findings from the Malignant Mesenchymal Tumour 89 (MMT 89) study [6]. The Children’s Oncology Group (COG), on the other hand, recommends a more aggressive approach to RT, citing high failure rates associated with its omission in these settings (albeit with reduced dosing of alkylator used in the MMT 89 study) [5]. This study examines the clinical profile, prognostic factors and outcomes of paediatric GU-RMS treated with a multimodal protocol at a single centre. The primary objective was to assess the EFS and OS, while the secondary objectives included evaluation of clinical characteristics and identification of prognostic factors impacting survival.

## Methods

This retrospective study adhered to the principles of the Declaration of Helsinki and Good Clinical Practice guidelines, receiving approval from the Institutional Ethics Committee. Children aged ≤ 15 years with biopsy-confirmed, treatment-naïve GU-RMS diagnosed between January 2013 and June 2022 were included. Clinical and treatment data were retrieved from institutional records. Staging included Fluorodeoxyglucose-Positron Emission Tomography Contrast Enhanced Computed Tomography (18F-FDG-PET CECT) and bilateral bone marrow aspiration and biopsy for earlier cohorts. Magnetic resonance imaging was utilised to enhance tumour delineation as needed. For bladder and vaginal primaries, diagnostic examination under anaesthesia and cystoscopy were performed. Patients underwent a standardised chemotherapy regimen comprising 12 cycles of vincristine, ifosfamide, etoposide (VIE) and/or vincristine, cyclophosphamide, dactinomycin (VAC) at 3-week intervals. Younger patients (<1 year) and those with obstructive uropathy received VAC to mitigate nephrotoxicity risks [7]. Cyclophosphamide was partially replaced with ifosfamide in earlier cohorts to minimise the cumulative toxicity of both. In later cohorts, VAC was adopted exclusively, given its suitability for outpatient administration (detailed in a previous paper) [7]. Fusion status (PAX-3/PAX-7 translocations) was analysed by Reverse Transcriptase Polymerase Chain Reaction, where available.

Local therapy commenced 10–12 weeks post-induction chemotherapy as determined by a multidisciplinary team. Modalities included RT, surgery or a combination, tailored to achieve R0 resection. Definitive RT to the primary tumour was delivered at a dose of 50.4 Gray in 28 fractions, utilising Intensity Modulated Radiation Therapy for precision. In the adjuvant setting, a reduced dose of 41.4 Gray was administered. Metastatic sites, including bone and lymph nodes, were addressed using similar RT doses. Lung metastases were reassessed after four cycles of induction chemotherapy. If lesions were in complete remission, patients were observed. If lesions had significantly reduced in size and were limited in number, metastatectomy was performed if feasible. For lung lesions deemed too small for surgery or those with multiple lesions showing substantial response, a repeat non-contrast chest CT scan was conducted after completing chemotherapy and was considered for whole lung irradiation on a case-to-case basis after discussion in the multidisciplinary tumour board.

Patients who underwent definitive RT had an 18F-FDG-PET CECT scan performed 3 months post-RT to evaluate disease status. They were further categorised as no residual disease, morphological residual disease (presence of a soft tissue lesion with no FDG avidity) or FDG-avid residual disease (any FDG activity in the residual lesion, regardless of grade).

### Statistical analysis

Baseline variables were analysed by descriptive statistics. For survival analysis, an event was defined as relapse, progression, treatment abandonment (started treatment and defaulted prior to completion of therapy), second malignancy or death due to any cause. Loss to Follow-Up was defined as a lack of follow-up for at least 18 months after completion of treatment. EFS and OS were calculated as time from the date of diagnosis to the event/last follow-up or death due to any cause/last follow-up, respectively. All patients without an event were censored at the last follow-up. Estimates of survival were computed using the Kaplan–Meier method. The hazard ratios (HRs) and significance associated with patient characteristics were assessed in a Cox proportional hazards regression model. Variables with a *p* value of <0.1 on univariate analysis were included in multivariate analysis, on which a *p* value of ≤ 0.05 was considered significant. Statistical analysis was performed using STATA software, version 15.1. An optimal cut-off for tumour size (tsize) with respect to EFS and OS was chosen in this study for outcome analysis. We optimised the cutoff by maximising the significance assessed by the log-rank test for the whole cohort. Kaplan–Meier method, which was used for the above optimisation analysis, was executed using the function ‘survfit’ from the R package ‘survival’.

## Results

### Epidemiological and clinical profile

**Figure 1.**
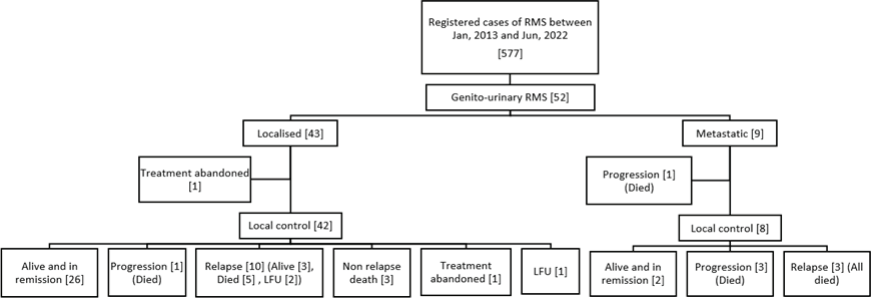
Consort diagram of this retrospective study.

During the study period, 52/577 (9 %) of patients diagnosed with RMS had GU primary. The median age at diagnosis was 3.1 years (range, 1.9–7.2 years) with a male-to-female ratio of 6.4:1. Nine patients (17.3%) had metastatic disease at presentation. The median tsize was 5.5 cm (range, 3.4–9.2 cm) and the median size of the regional lymph-node in involved cases was 1.7 cm (range, 1.0–2.7 cm). Detailed clinical characteristics are provided in Table 1. Consolidated Standards of Reporting Trials (CONSORT) diagram in Figure 1.

### Treatment

Two patients had events before local control (abandoned treatment-1, progression-1). For the remaining 50 patients, local treatment was definitive radiotherapy in 25 (49%), only surgery in 11 (21.5%-paratesticular (High inguinal orchiectomy) = 8, prostate (pelvic mass excision) = 1, bladder (radical cystectomy) = 1, urachus (excision) = 1) and combined (surgery + RT) in 14 (26.9%- Paratesticular (High Inguinal orchiectomy) = 6, Bladder dome (partial cystectomy, radical cystectomy with ileal conduit) = 2, Bladder neck (radical cystectomy with ileal conduit) = 2, Vagina (mass excision) = 2, Labia (wide excision) = 1 and Urachus (excision) = 1) patients. Surgical resection was R0 (*n* = 16), R1 (*n* = 1) or R2 (*n* = 4). Surgical details were not available in four patients who were operated upfront outside the institution (None received RT; two had metastatic disease and progressed while on chemotherapy, one had Group I disease and the fourth died due to sepsis). Median time to any local therapy for the primary was 14.5 weeks (range, 0–19.9 weeks). Twenty children received local therapy before 10–12 weeks (Para testicular = 13, Bladder =5, Prostate =1, Vagina = 1), reasons being: Prostate-operated upfront outside and presented to us with metastatic disease and progressed on chemotherapy; Vagina-Surgery (R0) at 12 weeks, followed by adjuvant radiotherapy; Bladder 1-definitive RT at 10 weeks; Bladder 2-definitive RT at 10.8 weeks; Bladder 3-upfront outside operated, died of urosepsis while on chemotherapy at our centre; Bladder 4 and 5-early surgery at 6 and 7 weeks, respectively (followed by adjuvant RT), as both had presented with bladder outlet obstructions (one with suprapubic cystostomy, the other with bilateral pervutaneous nephrostomies), complicated by recurrent urinary tract infections compromising delivery of chemotherapy. Median time to RT was 20.2 weeks (range, 17.4–24.1 weeks). Brachytherapy was used in two patients (bladder dome RMS = 1 and labial RMS = 1).

Nine patients had presented with metastatic disease. Surgical clearance for metastases was utilised in two patients (Pulmonary metastatectomy = 1 and infracolic omentectomy = 1). Four patients had disease progression, of whom three did not receive therapy for the metastases (opted for best supportive care) while one progressed during administration of definitive RT. For the rest, metastases were addressed with RT according to the site (whole lung irradiation = 3).

### Outcomes

At the time of analysis, 31 patients were alive, 16 had died, two had abandoned treatment and three were lost to follow-up. Disease-related mortality was 25% (*n* = 13). Non-relapse mortality was 5.7% (*n* = 3, sepsis-2 and urosepsis-1). There were 23 events in the cohort (relapse-13, progression-5, abandonment-2 and non-relapse deaths-3). Four of the 13 relapses were salvaged with three long-term survivors and one mortality due to therapy-related acute myeloid leukaemia. Two patients abandoned treatment, one post induction chemotherapy just before local therapy was to be delivered and the other after having completed local treatment with RT, while on adjuvant chemotherapy.

**Figure 2.**
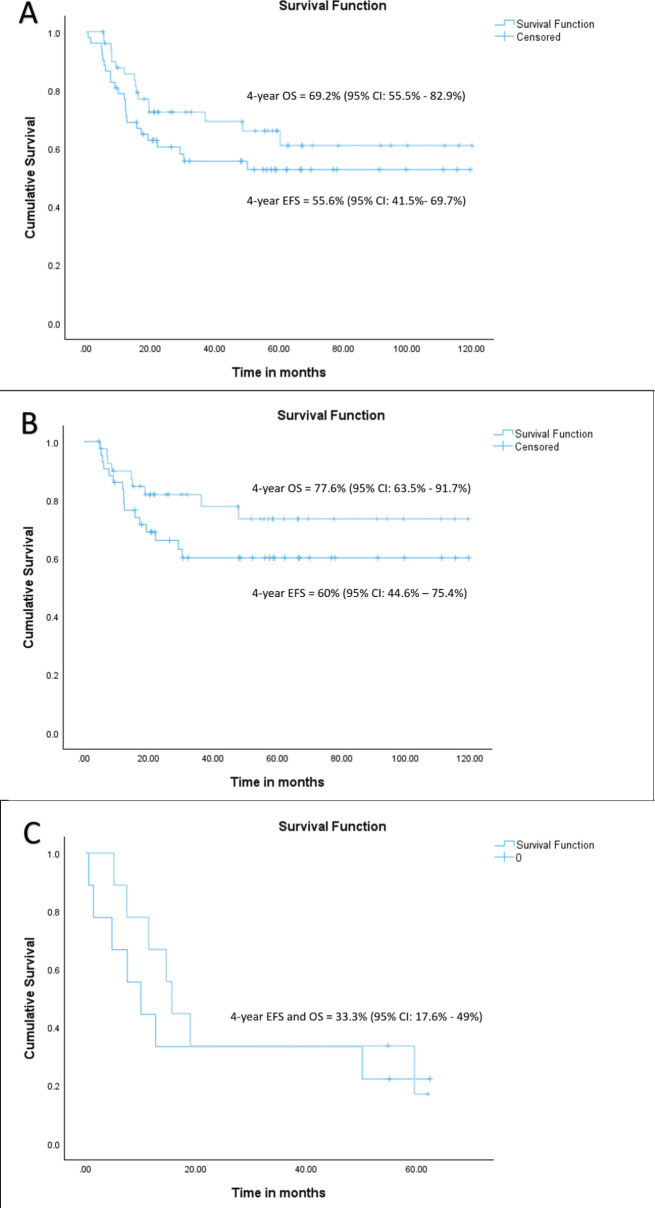
(a) EFS and OS of whole cohort, (b) EFS and OS of localised cohort and (c) EFS and OS of metastatic cohort.

**Figure 3.**
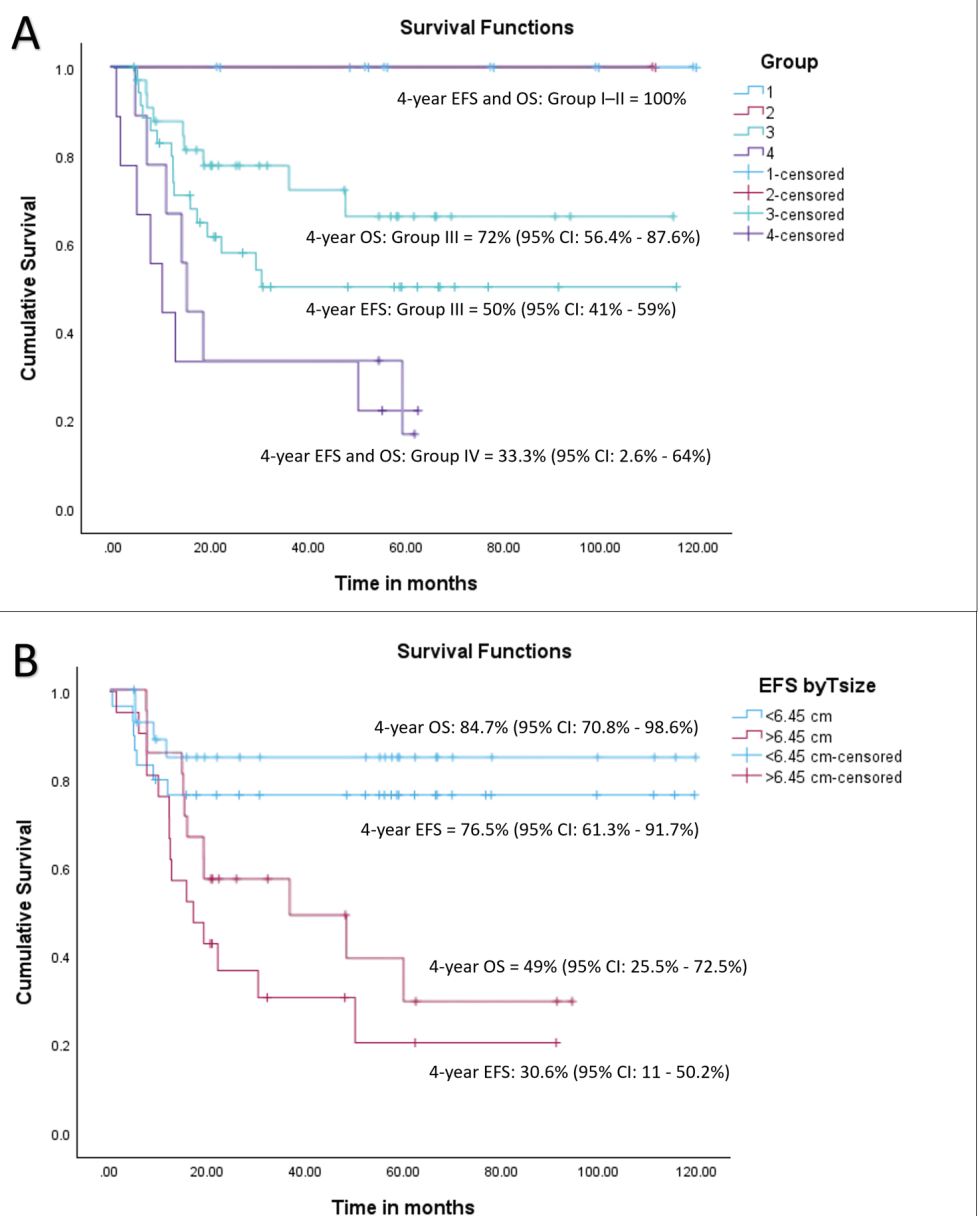
(a) EFS and OS of whole cohort by Group and (b) EFS and OS of whole cohort by tsize.

At a median follow-up of 56 months (95% confidence interval (CI): 49.1%–63.1%), 4-year EFS and OS of the whole cohort were 55.6% (95% CI: 41.5%–69.7%) and 69.2% (95% CI: 55.5%–82.9%), respectively. Among patients with localised disease, the 4-year EFS and OS were 60% (95% CI: 44.6%–75.4%) and 77.6% (95% CI: 63.5%–91.7%), respectively. For those with metastatic disease, both the 4-year EFS and OS were 33.3% (95% CI: 17.6%–49%). When stratified by disease group, patients in groups I–II demonstrated a 4-year EFS of 100%, while the corresponding rates for Groups III and IV were 50% (95% CI: 41%–59%) and 33.3% (95% CI: 2.6%–64%), respectively (*p* = 0.01). The corresponding 4-year OS rates for groups I–II, III and IV were 100%, 72% (95% CI: 56.4%–87.6%) and 33.3% (95% CI: 2.6%–64%), respectively (*p* = 0.007). According to risk stratification, patients belonging to low-risk and high-risk RMS demonstrated 4-year EFS and OS of 100% and 20% (95% CI: 1%–55%), respectively. The corresponding values for intermediate risk RMS were 51.9% (95% CI: 35.7%–68%) and 69.8% (95% CI: 53.6%–86%) [8]. Both the EFS and OS were highly significant across the three risk groups (*p* = 0.008 and *p* = 0.004, respectively). Further details are presented in Table 2 and survival curves are illustrated in Figures. 2a–c and 3a,b.

A total of 13 children relapsed, while five progressed on therapy. Relapses were locoregional (*n* = 4, 30.7%), metastatic (*n* = 5, 38.6%) and combined (*n* = 4, 30.7%). The median time to relapse was 15.7 months (range, 12.2–25.6 months). Of these, 11 relapses (84.6%) were early (≤24 months Disease-free Interval (DFI)), while 2 (15.4%) were late relapses (>24 months DFI).

**Figure 4.**
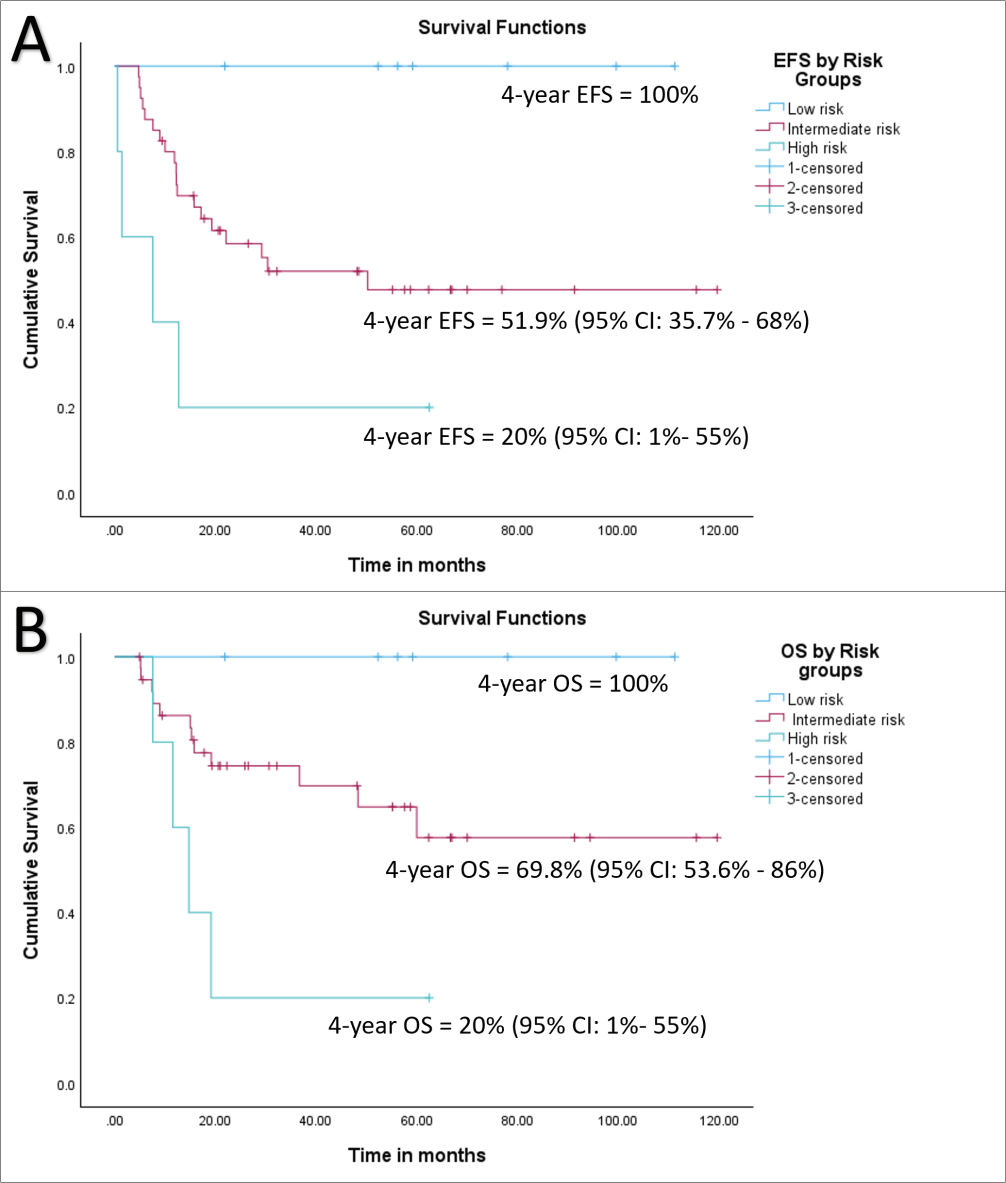
(a) EFS of whole cohort by risk group and (b) OS of whole cohort by risk group.

A tsize threshold of 6.45 cm emerged as a prognostic marker for outcomes, differing from the 5 cm threshold used in cooperative group studies [9]. For the entire cohort, the 4-year EFS rates were 76.5% (95% CI: 61.3%–91.7%) for tumours < 6.45 cm and 30.6% (95% CI: 11–50.2%) for tumours > 6.45 cm (HR = 3.6, 95% CI: 1.45–8.98, *p* = 0.006). Among patients with localised disease, the corresponding 4-year EFS rates were 81.3% (95% CI: 67.6%–95%) and 28% (95% CI: 4.5%–51.5%) (HR = 4.1, 95% CI: 1.38–12.1, *p* = 0.01). Similarly, the 4-year OS rates for the entire cohort were 84.7% (95% CI: 70.8%–98.6%) for tumours < 6.45 cm and 49% (95% CI: 25.5%–72.5%) for tumours > 6.45 cm (HR = 4.38, 95% CI:1.4–13.7, *p* = 0.01). For the localised cohort, the corresponding 4-year OS rates were 91.5% (95% CI: 80.1%–100%) and 53.3% (95% CI: 23.9%–82.7%), (HR = 6.93, 95% CI: 1.4–34.1, *p* = 0.017), respectively. Details are tabulated in Table 2. Survival curves are presented in Figures 3a,b and 4a,b.

The 4-year EFS rate for bladder/prostate (BP) primaries within the entire cohort was 48.5% (95% CI: 29.3%–67.7%) compared to 64.2% (95% CI: 44.4%–83.8%) for non-BP (non-BP) primaries (HR = 1.46, 95% CI: 0.63–3.38, *p* = 0.37). Among patients with localised disease, the 4-year EFS rates were 52% (95% CI: 32.4%–71.6%) for BP primaries and 70.1% (95% CI: 50.5%–89.7%) for non-BP primaries (HR = 1.9, 95% CI: 0.66–5.5, *p* = 0.22). The 4-year OS rate for BP primaries within the entire cohort was 63.8% (95% CI: 44.2%–83.4%) compared to 69.3% (95% CI: 49.7%–88.9%) for non-BP primaries (HR = 1.12, 95% CI: 0.4–3.1, *p* = 0.8). For the localised cohort, the corresponding 4-year OS rates were 71% (95% CI: 51.4%–90.6%) for BP primaries and 87.5% (95% CI: 71.8%–100%) for non-BP primaries (HR = 1.6, 95% CI: 0.4–6.4, *p* = 0.49). Details are presented in Table 2.

Among the 25 children who underwent definitive RT as local therapy, post-RT 18F-FDG-PET CECT scans were available for 17 patients. The remaining eight patients either had not reached the 3-month post-RT time point due to disease progression or were treated in earlier years when PET scans were not routinely performed. Of the 17 patients with available scans, 8 (47.0%) showed no residual disease, 5 (29.4%) had anatomical residuals and 4 (26.6%) had FDG-avid residuals. Among the four patients with FDG-avid residual disease, two experienced relapse and subsequently died (local relapse = 1 and metastatic relapse = 1). The 4-year EFS rate for the entire cohort based on PET response was 50% (95% CI: 1%–99%) for those with FDG-avid residuals and 75% (95% CI: 51.5%–98.5%) for those with no residual or anatomical residuals combined (*p* = 0.15). The corresponding OS rates were 50% (95% CI: 1%–99%) for FDG-avid residuals and 100% for the no residual/anatomical residual group (*p* = 0.009).

### Prognostic factors

For the entire cohort, univariate analysis identified metastatic disease (HR = 3.2, 95% CI: 1.3–7.8, *p* = 0.019), time to the first modality of local therapy (HR = 1.05, 95% CI: 1.00–1.10, *p* = 0.037) and tumour size > 6.45 cm (HR = 3.6, 95% CI: 1.45–8.98, *p* = 0.006) as prognostic for EFS. However, on multivariate analysis, only tumour size > 6.45 cm remained significant (HR = 3.99, 95% CI: 1.35–11.8, *p* = 0.012). For OS, univariate analysis revealed metastases (HR = 4.46, 95% CI: 1.65–12, *p* = 0.003), tumour size > 6.45 cm (HR = 4.38, 95% CI: 1.4–13.7, *p* = 0.01) and post-RT PET FDG-avid residual disease (*p* = 0.009) as significant prognostic factors. On multivariate analysis, only tumour size > 6.45 cm remained significant (HR = 3.8, 95% CI: 1.1–12.7, *p* = 0.031). Details are provided in Tables 3–5.

For the localised cohort, univariate analysis identified Group III disease (*p* = 0.003), post-RT PET FDG-avid residual disease (HR = 9.35, 95% CI: 0.84–103.9, *p* = 0.06), time to first local therapy (HR= 1.08, 95% CI: 1–1.1, *p* = 0.01) and tsize > 6.45 cm (HR = 4.1, 95% CI: 1.38–12.1, *p* = 0.01), to be prognostically significant for EFS. Delay in RT of > 20.5 weeks (HR = 3.34, 95% CI: 0.89–12.4, *p* = 0.07) showed a trend toward poor EFS. For OS, univariate analysis showed tsize > 6.45 cm (HR = 6.93, 95% CI: 1.4–34.1, *p* = 0.017) and post-RT PET FDG-avid residual disease (*p* = 0.009) as significant prognostic factors. Neither the type of local therapy across all cohorts (*p* = 0.8 and *p* = 0.5 for the whole and localised cohorts, respectively), nor the time to the first modality of local therapy for Group III tumours (EFS: HR = 1.05, 95% CI: 0.98–1.1, *p* = 0.13; OS: HR = 1, 95% CI: 0.92–1.07, *p* = 0.98) was prognostically significant. Multivariate analysis for the localised cohort could not be performed due to the small sample size and limited events. Details are presented in Tables 3, 4 and 6.

## Discussion

The EFS (55.6%) and OS (69.2%) observed in our study are notably inferior to outcomes reported in North American (Intergroup Rhabdomyosarcoma Study (IRS)-IV-EFS 76%, OS 80%) and European Paediatric Soft-tissue Sarcoma Study Group studies (EpSSG 2005-EFS 70.7%, OS 80.4%), despite the administration of local treatment in all of our patients [9, 10]. This disparity may be attributed to the unique characteristics of our cohort, including a higher proportion of large tumours at presentation (55.7% with *t* > 5 cm and 40.4% with *t* > 6.45 cm compared to 49% with *t* > 5 cm in SIOP MMT-89 and IRS-IV), a higher frequency of unfavourable primary sites (55.7% BP in our study versus 40% from the Surveillance, Epidemiology and End Results database) [5] and predominance of group III disease as opposed to more localised tumours from the American and European cohorts (67.3% Group III in our study compared to 30% in IRS-IV and 33% in SIOP MMT-89) [6, 9].

The EFS for Group III disease in our cohort was particularly poor, with 48% and 62%, respectively, for BP and non-BP primaries, compared to 73% (IRS-IV) and 63% (SIOP MMT-89) for BP primaries and 83% (IRS-IV) and 82% (SIOP MMT-89) for non-BP GU-RMS [6, 9]. This occurred despite the use of optimal chemotherapy, including cyclophosphamide at 2.2 gm/m^2^ per cycle or equivalent dosing of ifosfamide in earlier cohorts, as well as comprehensive local therapy for all patients. These outcomes contrast with prior studies from the SIOP MMT series, where local therapy was reserved for those who could not achieve a complete response with induction chemotherapy, yielding a lower EFS (63%). Regardless, the OS (77%) was comparable to that of other international trials, with 50% of survivors not requiring local therapy, at the expense of intensified chemotherapy and the need for salvage options [11]. Moreover, the EpSSG has recently reclassified BP sites as favourable, which does not align with the outcomes observed in our cohort [12]. In the COG ARST 0531 trial, reducing the cumulative cyclophosphamide dose by 50% for intermediate-risk RMS decreased the toxicities but increased local failure rates [13]. However, the higher dose of cyclophosphamide used in our cohort was not associated with increased non-relapse mortality. Sepsis-related deaths were comparable to those in our other dose-intensive protocols, reflecting the background infection-related mortality in our institution. Our cohort's poorer EFS, particularly for Group III tumours across BP and non-BP primaries, may be driven partly by the predominance of locally advanced, unfavourable-site primary tumours with larger baseline sizes. Tsize at diagnosis emerged as a significant prognostic factor for both EFS and OS, albeit at a higher cutoff (6.45 versus 5 cm in Western studies) [9]. An analysis of local therapy for primary tumours did not identify either the time-point or type of local therapy as a significant contributor to inferior Group III outcomes. However, a delay in RT beyond 20.5 weeks showed a trend toward poorer EFS in the localised cohort. Treatment deintensification strategies, as practiced in the Western world, appear unsuitable for our cohort at this juncture, particularly for Group III tumours, given the unique challenges and tumour biology observed in this population. Multi-centre studies incorporating molecular analysis are crucial to understanding the biology of Group III GU-RMS in our region and optimising outcomes, as this subgroup dominates such cases here.

A recent study from our institute reported a 3-year EFS of 58% for localised RMS across all sites. The 5-year EFS for localised tumours at favourable and unfavourable sites were 67.5% and 53%, respectively. However, as this study did not perform site-specific survival analysis, further deductions are limited, although the outcomes were relatively inferior compared to those in Western cohorts [14]. Another retrospective study spanning two decades from a tertiary cancer centre in India highlighted significantly poorer outcomes for BP RMS, with a 5-year EFS of 22.2% ± 1.2% (*p* = 0.03), compared to other favourable sites [15]. Additionally, a regional study using a lower cyclophosphamide dose (1.8 g/m² per cycle) reported a substantially inferior 3-year EFS of 14% for intermediate-risk RMS [16]. Similar findings were reported in a single-centre study from South Africa, which treated patients with RMS using IRS protocols. This study, conducted in a setting comparable to low-and middle-income-countries (LMICs), demonstrated a predominance of Group III disease (59%) and an OS of 65%, aligning with the trends observed in our current study [17]. While these retrospective analyses consistently show relatively inferior outcomes for RMS overall, site-specific outcomes for genitourinary RMS remain unavailable across these studies.

Our analysis reveals inferior 3-year survival outcomes compared to the earlier study from our institute [14]. Given that relapses in these patients are challenging to manage—especially due to the difficulty in re-administering local therapy in anatomically complex and critical regions—it is imperative to focus on delivering optimal therapy during the upfront treatment phase. This approach is particularly crucial in the LMIC context, where the burden and resource demands of treating relapses can be prohibitive.

Although the site of the primary tumour was not found to be a significant prognostic factor in this study—likely due to the small sample size—the tsize of 6.45 cm emerged as a significant prognostic indicator. As previously mentioned, this threshold was consistent across sites and stages, differing from the widely published cutoff of tsize > 5 cm [9]. This finding warrants prospective validation in future studies and holds potential for use in risk stratification in the LMIC setting. Identifying tumours exceeding this size threshold could aid in tailoring treatment approaches, including dose intensification, incorporation of targeted therapies, delayed primary excision (when feasible) and higher radiation doses, potentially employing advanced modalities like proton therapy or brachytherapy. However, these strategies need to be investigated further within the framework of clinical trials.

The outcomes for metastatic disease in our cohort were poor, with both 4-year EFS and OS at 33.3%, aligning with global data [2, 4]. This underscores the necessity of managing such high-risk groups within the context of clinical trials to explore innovative therapeutic strategies and improve survival outcomes.

Limitations of this study include its retrospective nature and a small sample size, which preclude conclusive negation of the prognostic significance of the site of primary and other factors. Also, fusion status is not available for all and the sequelae of local therapy are not addressed in this analysis. Despite these shortcomings, this study provides a single-centre experience of GU-RMS, which is rare in childhood and can help plan future studies on a multi-centre platform in the country.

## Conclusion

Outcomes of group III GU-RMS in children treated on a multimodal protocol in our study are suboptimal compared to those from co-operative group trials, probably affected by large tumours at presentation, warranting alternative strategies for optimisation of survival.

## List of abbreviations

ARMS, Alveolar Rhabdomyosarcoma; B, Bladder; CECT, Contrast Enhanced Computed Tomography; COG, Children’s Oncology Group; CONSORT, Consolidated Standards of Reporting Trials; DFI, Disease-free Interval; EFS, Event-Free Survival; EpSSG, European paediatric Soft tissue sarcoma Study Group; ERMS, Embryonal Rhabdomyosarcoma; FDG-PET, Fluorodeoxyglucose-Positron Emission Tomography; GU-RMS, Genitourinary-Rhabdomyosarcomas; IRS, Intergroup Rhabdomyosarcoma Study; LMIC, Low-and Middle-Income-Country; MMT89, Malignant Mesenchymal Tumour; OS, Overall Survival; P, Prostate; RT, Radiotherapy; SEER, Surveillance, Epidemiology and End Results; SIOP, International Society of Paediatric Oncology; Tsize, Tumour Size; VAC, Vincristine, cyclophosphamide, dactinomycin; VIE, Vincristine, ifosfamide, etoposide.

## Conflicts of interest

The authors declare that there are no conflicts of interest.

## Funding

Nil.

## Author contributions

Doctors Chakraborti and Parambil conceptualised and designed the study, conducted data analysis and interpretation and drafted the manuscript. Doctors Gollamudi, Prasad, Laskar, Khanna, Manjali, Qureshi, Ramadwar, Panjwani, Baheti, Patil, Shah and Chinnaswamy reviewed the manuscript and contributed to the final version with their insights and revisions.

## Figures and Tables

**Table 1.** Demographic and clinical characteristics of patients in this cohort.

Demographic or clinical characteristic	Number (%)	
	Entire cohort, *n* = 52	Localised cohort, *n* = 43
Gender		
Male	45 (85.0)	37 (86)
Age in years		
≤1	3 (5.7)	3 (6.9)
1–10	38 (73.0)	34 (79)
≥10	11 (21.0)	6 (14.1)
Regional lymph-node		
Positive	20 (38.5)	16 (37.0)
Radiology only	17 (85.0)	13 (81.2)
Fine needle aspiration cytology/biopsy	2 (10.0)	2 (12.5)
Combined (radiology + biopsy)	1 (5.0)	1 (6.3)
Group		
Group I	7 (13.4)	7 (16.3)
Group II	1 (1.9)	1 (2.3)
Group III	35 (67.3)	35 (81.4)
Group IV	9 (17.3)	0
Stage		
Stage 1	17 (32.7)	17 (39.5)
Stage 2	7 (13.4)	7 (16.3)
Stage 3	19 (36.5)	19 (44.2)
Stage 4	9 (17.3)	0
Risk		
Low	7 (13.4)	7 (16.3)
Intermediate	40 (76.9)	36 (83.7)
High	5 (9.6)	0
Histology	*n* = 51	*n* = 42
ERMS	47 (92.1)	40 (95.2)
ARMS	4 (7.8)	2 (4.8)
Fusion status	*n* = 35	*n* = 29
Fusion positive	3 (8.6)	3 (10.3)
PAX-3 positive	3 (8.6)	3 (10.3)
PAX-7 positive	0	0
Site		
Bladder	19 (36.5)	18 (41.9)
Prostate	10 (19.2)	7 (16.3)
Paratesticular	15 (28.8)	13 (30.2)
Vagina	4 (7.7)	3 (7)
Urachus	2 (3.8)	1 (2.3)
Others	3 (5.8)	1 (2.3)
Tumour size	*n* = 51	*n* = 42
³6.45 cm	21 (41.2)	15 (35.7)
Type of chemotherapy		
VIE + VCD	11 (21.1)	6 (14)
VCD	41 (77.3)	37 (86)
Delay in RT to > 20.5 weeks	*n* = 40	*n* = 33
	16 (40)	14 (42)
Residual in PET scan done post 3 months definitive RT	*n* = 17	*n* = 15
No residual	8 (47.0)	8 (53.3)
Morphological only residual	5 (29.4)	4 (26.7)
Metabolic residual	4 (23.6)	3 (20)

ARMS, Alveolar Rhabdomyosarcoma; ERMS, Embryonal Rhabdomyosarcoma; RT, Radiotherapy; VAC, Vincristine, Cyclophosphamide, Dactinomycin; VIE, Vincristine, Ifosfamide, Etoposide.

**Table 2.** EFS and OS comparisons by subsets within the cohort.

Variable	4-year EFS (%)	95% CI	*p* value	4-year OS (%)	95% CI	*p* value
Overall cohort	55.6	41.5–69.7		69.2	55.5–82.9	
Localised	60	44.6–75.4		77.6	63.5–91.7	
Metastatic	33.3	17.6–49		33.3	17.6–49	
Group I–II	100			100		
Group III	50	41–59		72	56.4–87.6	
Group IV	33.3	2.6–64	0.01	33.3	2.6–64	0.007
Whole cohort						
BP	48.5	29.3–67.7		63.8	44.2–83.4	
Non- BP	64.2	44.4–83.8	0.37	69.3	49.7–88.9	0.8
Localised cohort						
BP	52	32.4–71.6		71	51.4–90.6	
Non- BP	70.1	50.5–89.7	0.22	87.5	71.8–100	0.49
Whole cohort						
tsize < 6.45 cm	76.5	61.3–91.7		84.7	70.8–98.6	
tsize ≥ 6.45 cm	30.6	11–50.2	0.003	49	25.5–72.5	0.006
Localised cohort						
tsize < 6.45 cm	81.3	67.6–95		91.5	80.1–100	
tsize > 6.45 cm	28	4.5–51.5	0.006	53.3	23.9–82.7	0.006

**Table 3.** Univariate analysis- EFS of whole, localised and metastatic cohorts.

Variable	HR	95% CI	*p* value	HR	95% CI	*p* value	HR	95% CI	*p* value
	For whole cohort (*n* = 52)			For localised cohort (*n* = 43)			For metastatic cohort (*n* = 9)		
Gender (Male versus Female)	1.2	0.4–3.6	0.7	2.1	0.69–6.6	0.18	3.35	0.40–27.9	0.3
Age (>10 years versus £10 years)	1.85	0.76–4.5	0.17	1.25	0.39–4.4	0.7	1.58	0.35–7.1	0.55
Site of primary (BP versus others)	1.46	0.63–3.38	0.37	1.9	0.66–5.5	0.22	1.17	0.26–5.3	0.83
Histology (ARMS versus ERMS)	1.53	0.36–6.59	0.56	-	-	-	-	-	-
Lymph node involvement	2.08	0.9–4.7	0.08	1.69	0.63–4.5	0.29	5.4	0.95–30.8	0.057
Group (IV versus localised)	3.2	1.3–7.8	0.019	-	-	-	-	-	-
Radiotherapy > 20.5 weeks from start of chemotherapy	2.26	0.8–6.4	0.123	3.34	0.89–12.4	0.07	0.71	0.06–8	0.78
tsize > 5 cm	2.22	0.86–5.74	0.09	2.5	0.8–8.1	0.11	0.88	0.17–4.5	0.87
tsize > 6.45 cm	3.6	1.45–8.98	0.006	4.1	1.38–12.1	0.01	0.87	0.17–4.5	0.87
Post definitive radiotherapy PET FDG-avid residual	-	-	0.2	9.35	0.84– 103.9	0.06	-	-	0.4
Local therapy (RT versus Surgery)	0.88	0.25–3.14	0.8	1.7	0.33–8.79	0.5	0.04	0.00–0.78	0.034

**Table 4.** Univariate analysis- OS of whole, localised and metastatic cohorts.

Variable	HR	CI	*p* value	HR	CI	*p* value	HR	CI	*p* value
	For whole cohort			For localised cohort			For metastatic cohort		
Gender (male versus female)	2.59	0.34–19.7	0.35	1.32	0.16–10.6	0.78	2.84	0.33–24.4	0.3
Age (>10 years versus £10 years)	2.38	0.86–6.5	0.09	1.67	0.35–8.1	0.52	1.23	0.27–5.5	0.8
Site of primary (BP versus others)	1.12	0.4–3.1	0.8	1.12	0.4–3.1	0.8	0.92	0.2–4.2	0.9
Histology (ARMS versus ERMS)	2.9	0.6–13	0.16	-	-	-	3.38	0.03–1.81	0.2
Lymph node involvement	3.36	1.2–9.4	0.02	3.75	0.9–15.1	0.06	4.1	0.7–23.4	0.1
Group (IV versus localised)	4.46	1.65–12	0.003	-	-	-	-	-	-
Radiotherapy > 20.5 weeks from start of chemotherapy	1.01	0.27–3.8	0.98	1.07	0.2–5.3	0.93	1.22	0.1–19.8	0.89
tsize > 5 cm	2.4	0.77–7.6	0.12	3.23	0.65–15.8	0.15	0.65	0.12–3.62	0.62
tsize > 6.45 cm	4.38	1.4–13.7	0.01	6.93	1.4–34.1	0.017	0.65	0.11–3.62	0.6
Post definitive radiotherapy PET FDG-avid residual	-	-	0.009	-	-	0.009	-	-	0.6
Local therapy (RT versus surgery)	0.91	0.26–3.23	0.9	1.71	0.33–8.82	0.5	0.06	0.00–1.17	0.063

**Table 5.** Multivariate analysis- EFS and OS of whole cohort.

Variable	HR	CI	*p* value	HR	CI	*p* value
	For EFS			For OS		
**Group IV**	1.75	0.64–4.75	0.27	2.33	0.79–6.8	0.125
**Tumor size > 6.45 cm**	3.99	1.35–11.8	0.012	3.8	1.1–12.7	0.031

**Table 6.** Univariate analysis- EFS and OS for whole cohort and Group III by time to local therapy.

Variable	HR	CI	*p* value	HR	CI	*p* value
	For whole cohort (*n* = 51)			For Group III (*n* = 35)		
EFS						
First modality of local therapy ≥12 weeks from start of chemotherapy	1.53	0.59–3.9	0.38	1.25	0.39, 3.94	0.7
Time to first modality of local therapy	1.05	1.00, 1.10	0.037	1.05	0.98, 1.1	0.13
OS						
First modality of local therapy ≥12 weeks from start of chemotherapy	0.97	0.33–2.8	0.96	0.64	0.16, 2.58	0.53
Time to first modality of local therapy	1.01	0.96, 1.07	0.49	1	0.92, 1.07	0.98
